# Describing socioeconomic gradients in children’s diets – does the socioeconomic indicator used matter?

**DOI:** 10.1186/1479-5868-11-44

**Published:** 2014-03-28

**Authors:** Dorota Zarnowiecki, Kylie Ball, Natalie Parletta, James Dollman

**Affiliations:** 1 School of Population Health, University of South Australia, GPO Box 2471, Adelaide, SA 5001, Australia; 2 School of Nutrition and Exercise Science, Deakin University, Burwood Highway, Burwood, VIC3125, Australia; 3 School of Health Sciences, University of South Australia, GPO Box 2471, Adelaide, SA 5001, Australia

**Keywords:** Socioeconomic position, Children, Diet, Fruit, Vegetables, Non-core food, Sweet drinks

## Abstract

**Background:**

Children of low socioeconomic position (SEP) generally have poorer diets than children of high SEP. However there is no consensus on which SEP variable is most indicative of SEP differences in children’s diets. This study investigated associations between diet and various SEP indicators among children aged 9–13 years.

**Method:**

Families (n = 625) were recruited from 27 Adelaide primary schools in 2010. Children completed semi-quantitative food frequency questionnaires providing intake scores for fruit, vegetables, non-core foods, sweetened drinks, and healthy and unhealthy eating behaviours. Parents reported demographic information by telephone interview. Differences in dietary intake scores were compared across parental education, income, occupation, employment status and home postcode.

**Results:**

Across most SEP indicators, lower SEP was associated with poorer dietary outcomes, including higher intake of non-core foods and sweetened drinks, and more unhealthy behaviours; and lower intake of fruit and vegetables, and fewer healthy behaviours. The number and type of significant SEP-diet associations differed across SEP indicators and dietary outcomes. Mother’s education appeared most frequently as a predictor of children’s dietary intake, and postcode was the least frequent predictor of children’s dietary intake.

**Conclusion:**

Socioeconomic gradients in children’s dietary intake varied according to the SEP indicator used, suggesting indicator-specific pathways of influence on children’s dietary intake. Researchers should consider multiple indicators when defining SEP in relation to children’s eating.

## Background

A relatively consistent body of literature shows that children and adolescents of low socioeconomic position (SEP) are at risk of consuming poorer diets, due to lower fruit and vegetable consumption [1-3], and higher intake of snack foods, fast foods and sweetened beverages [3-5]. These types of dietary patterns may contribute to higher energy and fat intake among children of low SEP [6], higher obesity rates [7], and higher rates of cardiovascular disease in later life [8]. The drivers of these SEP gradients in children’s diets are not well understood. One reason for this may be that it is unclear what aspects of SEP are most important or how different SEP indicators might be differentially associated with diet. A better understanding of this would give insights into potential mechanisms by which socioeconomic disadvantage leads to poorer dietary intake.

A range of SEP indicators have been used to identify associations of SEP with children’s diet. Parent education, occupation and income are the most frequently used SEP indicators [1,9]. In addition to these, SEP has been defined using census data as an indicator of neighbourhood disadvantage [4,10,11], receipt of reduced cost or free school meals [12,13], and perceived family affluence defined by factors such as internet access and car ownership [14,15]. Composite scores of SEP combining multiple socioeconomic indicators have also been used [16,17]. Most studies define SEP using one socioeconomic indicator (for example, [13,18,19]), however SEP is the product of a number of social and economic factors [20], and several indicators may be needed to more confidently characterise SEP disparities in children’s dietary intake [21].

Limited evidence suggests that SEP indicators may be both independently and synergistically associated with children’s diet. Vereecken et al. [22] simultaneously modeled the effects, firstly, of occupation and family affluence on adolescents’ fruit and soft drink intake, and secondly, the effect of family affluence, occupation and school-level affluence. Adolescents from families of higher affluence and parental occupation status consumed more fruit, and those with parents of high occupation status consumed less soft-drink. These gradients were maintained but slightly attenuated by the inclusion of the area-level measures in the final models, indicating that all three indicators independently contributed to explaining differences in children’s fruit and soft drink intake. Both maternal education and area-level SEP were independently related with “unhealthy eating” and “unhealthy snacking” patterns of 11-year old children, when included simultaneously in adjusted models [23]. The influence of SEP indicators may also differ for boys and girls. In multivariate models including seven SEP indicators, maternal education and family affluence independently predicted adolescent girls’ breakfast consumption, whereas only perceived family wealth predicted boys’ breakfast consumption [14]. Few other studies have simultaneously modeled associations of a number of socioeconomic indicators with diet in youth. Among adults, Lakulla and colleagues [21] modeled multiple SEP indicators in relation to a composite dietary intake score of healthy food habits in Finnish adults. In final models including all seven SEP indicators, significant associations remained between each indicator of low SEP and less healthy food habits; however these relationships were attenuated compared with initial independent models for SEP indices. Galobardes et al. [24] modeled interactions between education and occupation, showing a significant additive effect on men’s starchy carbohydrate consumption (pasta and potatoes), whereby consumption increased by education level within each occupation group, with highest consumption among men who were of both low occupation and low education.

These studies highlight the complexity of conceptualizing SEP in health research, and suggest that SEP indicators are not interchangeable but are each operationally distinct and may influence health behaviours by conceptually different processes [25]. While this issue has been considered in a number of studies of adults [21,24-26], there has been less consideration of how different SEP indicators influence children’s dietary intake. A better understanding of the independent contributions of various indicators of SEP to children’s diets will help shed light on both the most vulnerable target groups, and potential underlying mechanisms for health promotion. Therefore, the aim of this study was to investigate the strength and independence of the associations between children’s dietary intake and various indicators of SEP (education, occupation, employment status, income, and postcode).

## Methods

This was a cross-sectional study conducted in Adelaide, South Australia from March to November 2010, involving children aged 9–13 years and their parents. Ethics approval was obtained from the University of South Australia Human Research Ethics Committee and the Department of Education and Children’s Services Ethics Committee. Parents provided written consent for their family to participate in the study, and children provided verbal assent before commencing study measures.

### Participant recruitment

Participants were recruited from grades five to seven of Government primary schools in metropolitan Adelaide. In Australia, parents may choose to send children to government, independent or Catholic schools. In this study, we recruited from government schools which children from across all socioeconomic strata attend. Schools were divided into tertiles by SEP using the School Card Register (SCR), which ranks schools according to the percentage of students receiving means-tested Government assistance to meet the cost of school attendance. Schools were randomly selected from each tertile and study information was sent by email to the school Principal. Recruitment started in March 2010, and continued until September 2010 simultaneously with data collection. This allowed the number of participants completing the study from each SEP tertile to be monitored and more schools to be recruited from the required SEP tertile to ensure an even distribution of participants across socioeconomic strata. Eighty-four schools were sent information about the study, of which 27 agreed to participate (32% response rate). Ten of these schools were low SEP, six mid SEP and 11 high SEP according to the SCR, with a significantly lower response rate of 28% in low SEP schools (p < 0.000).

Participating schools distributed study information sheets and consent forms to parents of students in participating classes. Information was distributed to 2575 students, of which 1257 returned consent forms indicating consent to participate in the study (48.8% response rate), and 1201 students completed the study measures (95.6% of participating students). Children were excluded from participation if they had chronic conditions affecting dietary intake or seriously limiting their ability to complete study measures, and if they or their parents were unable to speak and understand English with sufficient fluency to complete study measures. Of the 1201 participants, 625 are included in this analysis as they had both complete food intake data (from phase 1) and parent-reported demographic information (from phase 2 CATI). Demographic information was not collected from participants who did not complete the CATI, and therefore it was not possible to analyse whether there were any differences between those and parents who are included in this analysis. However, children whose parents completed the CATI were significantly more likely to live in a neighbourhood of higher SEP (determined using the SEIFA index described below), but there was no difference in children’s age, sex or dietary outcomes.

### Procedure

The study was conducted over two phases: in the first phase children completed the Child Nutrition Questionnaire (CNQ), and in the second phase parents completed a ‘computer-assisted telephone interview’ (CATI). The CNQ was completed by children online using school computer facilities during school hours. Groups of 15 to 30 students were supervised during questionnaire completion by three to four research assistants. At completion of phase one, parents were invited by telephone call to complete the CATI if their child had complete survey data. The CATI was conducted by trained interviewers with the parent primarily responsible for their child’s food provision and lasted 15–20 minutes. During the CATI parents responded to questions about predictors of children’s health behaviours and demographic information about the family.

### Study measures

Children’s dietary intake was measured using the Child Nutrition Questionnaire (CNQ), a semi-quantitative food frequency questionnaire designed to measure dietary patterns associated with positive energy consumption [27]. The CNQ provided six scores of dietary intake and eating behaviours, capturing usual patterns of children’s consumption of fruits, vegetables, non-core foods, sweetened beverages and healthy and unhealthy eating behaviours. Dietary intake scores were formed by summing scores on individual responses, with questions about weekly intake recoded to represent daily intake for consistency between items. Details about questions, score structure and psychometric properties are provided in Table 1 and have been previously described [27]. Validity and reliability of the CNQ were determined to be acceptable by Wilson et al. [27], and similar to other instruments measuring dietary intake in this age group [28,29].

**Table 1:** Summary of questions and mean intake scores from Child Nutrition Questionnaire

**Scale**	**Items**	**Response**	**Score range**	**Target healthy value**^**#**^	**Boys (n = 275) Mean (SD)**	**Girls (n = 353) Mean (SD)**
Fruit intake (3-items)	(1) Consumption of fruit at recess/lunch/after school, (2) Variety of fruits consumed yesterday, (3) Usual serves of fruit per day	(1–2) Tick if consumed; (3) Frequency scale^a^	0 - 16	≥ 6	5.44 (2.92)	5.35 (2.62)
Vegetable intake (3-items)	(1) Consumption of vegetables at recess/lunch/after school, (2) variety of vegetables consumed yesterday, (3) usual serves of vegetables per day	(1–2) Tick if consumed; (3) Frequency scale^a^	0 - 13	≥ 8	4.72 (2.40)	4.71 (2.40)
Non-core food intake (2-items)	(1) Consumption of 11 non-core foods at recess/lunch/after school, (2) No. times/week non-core foods consumed^d^	(1) Tick if consumed; (2) Frequency scale^b^	0 - 33	≤ 1	3.56 (2.33)	3.69 (1.91)
Sweet drink intake (2-items)	(1) Consumption of sweet drinks (incl. juice) at recess/lunch/after school, (2) No. times/week sweet drinks consumed	(1) Tick if consumed; (2) Frequency scale^b^	0 - 14	≤ 1.3	1.75 (1.35)	1.67 (1.27)
Healthy behaviours (5-items)	No. times p/week: (1) Eat breakfast, (2) Carry water bottle, (3) Help with groceries, (4) Help prepare dinner, (5) Eat dinner with family	Frequency scale^c^	4 - 20	n/a	17.42* (3.57)	18.42* (3.68)
Unhealthy behaviours (3-items)	No. times p/week: (1) Eat dinner in front of TV, (2) Eat snacks in front of TV, (3) Eat fast food	Frequency scale^c^	3 - 15	n/a	7.64 (2.84)	7.68 (2.58)

*p = 0.001 for difference in dietary intake scores between boys and girls.

^#^Target healthy values established by Wilson et al. 2008 [27].

^a^Frequency scale (scoring in parentheses): I don’t eat fruit/vegetables (0.0), less than 1 serve per day (0.5), 1–2 serves per day (1.5), 3–5 serves per day (4.0), and more than 5 serves per day (6.0).

^b^Frequency scale (Weighted for daily consumption by dividing weekly score by seven - shown in parentheses): never/rarely (0.0), less than once/week (0.07), about 1–3 times per week (0.29), about 4–6 times per week (0.71), every day (1.00).

^c^Frequency scale: never/rarely, less than once/week, about 1–3 times per week, about 4–6 times per week, every day; scored 1–5 respectively.

^d^(1) 11 non-core food items measured were: potato crisps, chocolate, lollies, muesli bar, savoury biscuits, sweet biscuits, ice-cream/ice-block, hot chips, pie/pasty/sausage roll, hot dog, pizza; (2) Non-core foods consumed were: chocolate/lollies, potato crisps, hot chips.

Parents reported demographic information about mother’s education, occupation, household income, home postcode and parent age and marital status (used as covariates in data analysis). Maternal demographic characteristics were used as mothers are likely to be the gatekeepers to family food environments, and as such maternal socioeconomic factors may more directly impact children’s dietary habits than paternal socioeconomic factors [1,30]. Mother’s education level was reported on an eight point scale ranging from (1) never attended school to (8) completed postgraduate education. Parents reported mother’s job title, and this information was coded using the Australian and New Zealand Standard Classification of Occupations (ANZSCO) which groups occupations into eight hierarchical tiers according to level of skills, education, responsibility and experience required to perform the occupation [31]. An additional category was created for individuals ‘not in the labour force’, comprised of individuals engaged in full-time home duties, retired persons, unemployed and students. Where insufficient information was provided in the job title to accurately classify an individual, for example ‘public servant’, these data points were not coded and were treated as missing data. Additionally, mother’s employment status was dichotomised into employed, and not in the labour force. Gross household income per annum before tax, including pensions and government assistance, was reported using seven income brackets ranging from (1) Up to AU$12,000 to (7) More than AU$100,000. Home postcodes were classified into tertiles of SEP using the Socioeconomic Index for Areas (SEIFA) score of disadvantage, an area-level index of disadvantage based on census data for factors such as income, education and occupation (Australian Bureau of Statistics, 2008).

### Data analysis

Demographic information collected from parents was matched with children’s responses to the CNQ. If one or more items within a dietary outcome score were missing, the score for that participant was not calculated. Initial linear models were constructed, adjusted and unadjusted for clustering by class and school, finding no evidence of clustering of dietary variables in classes within schools. All analyses were conducted separately for boys and girls as SEP associations with diet may differ between boys and girls [18]. SPSS version 19 was used to generate descriptive statistics and conduct independent samples t-tests to compare differences in dietary intake between boys and girls.

Correlated component regression (CCR) was used to identify socioeconomic predictors of dietary outcomes. All SEP variables (mother’s education, income, mother’s occupation, employment status, and SEIFA) were entered simultaneously into the CCR model along with possible covariates of child age, mother’s age and marital status. CCR employs cross-validation with a step-down algorithm to reduce the number of predictors in the model, producing a linear combination of explanatory variables used to predict a function of the dependent variable [32]. The final CCR models reflect the predictors maximising the R-squared and variance explained by the predicting equation. CCR was used as it allows for simultaneous consideration of multiple SEP variables which have mixed scale types and may be correlated, as would be expected with variables representing SEP. Variable importance was compared using both standardized regression coefficients (*β*) and cross-validation predictor counts that reflect the number of occasions the variable appeared as a predictor in regression models. CCR analyses were conducted using XLSTAT 2012.

## Results

### Demographic and descriptive data

Complete dietary intake data and demographic information were collected from 625 child–parent dyads (Tables 1 and 2). The mean age of participating children was 11.3 years and 56% were girls. Participating families were well distributed across the socioeconomic strata for all SEP indicators. Table 1 presents the means and standard deviations for dietary intake scores stratified by sex. Dietary intake scores were similar for boys and girls except for the healthy behaviour score, which was significantly higher in girls. Comparison of dietary intake scores from the CNQ with dietary guidelines is limited. To enable evaluation of the CNQ dietary intake scores, target healthy values have been established for these scores based on Australian dietary guidelines as listed in Table 1[27]. Mean vegetable intake scores were considerably lower than the target healthy value, and non-core food intake scores were almost four times greater than the target value. Encouragingly, mean sweetened drink intake scores exceeded the target healthy value by only a small amount.

**Table 2:** Demographic characteristics of participants

	**All (n = 628)**	**Boys (n = 275)**	**Girls (n = 353)**
Child age (years)	11.3 (0.90)	11.4 (0.89)	11.3 (0.90)
School grade (%)			
Grade 5	26.2	26.6	26.0
Grade 6	39.9	38.3	41.2
Grade 7	33.9	35.0	32.8
Parent age (years)	42.1 (5.54)	42.3 (5.30)	42.0 (5.70)
Marital status (%)			
Partner	78.2	76.2	79.9
No partner	21.8	23.8	20.1
Mother’s education level^a^ (%)			
Never attended school	0.2	0.0	0.3
Some high school	22.9	22.3	23.4
Completed high school	19.1	18.2	19.8
Trade or diploma	26.9	24.5	28.5
University degree	19.2	22.3	16.9
Higher University degree	11.8	12.8	11.0
Gross household income^bc^ (%)			
Low	33.9	31.9	35.2
Mid	37.4	39.2	36.1
High	28.7	28.8	28.6
SEIFA^d^ (%)			
Low	33.5	31.8	35.0
Mid	31.8	32.8	30.8
High	34.7	35.4	34.2
Mother’s occupation^e, f^ (%)			
Managers	7.0	5.9	7.8
Professionals	22.5	24.8	20.8
Technicians and trades	4.4	4.8	4.0
Community and personal service	11.5	12.2	11.0
Clerical and administrative	21.6	20.7	22.0
Sales	4.7	6.7	3.2
Machinery operators and drivers	0.3	0.4	0.3
Labourers	4.9	5.9	4.0
Not in the labour force	21.2	17.4	24.3
Mother’s employment (%)			
Not in the labour force	21.7	17.6	24.9
Employed	78.3	82.4	75.1

Data reported as percentage (%) where indicated, or as mean (standard deviation).

^a^Education level measured on an 8-point scale (never attended school – higher university degree) – no participants recorded for responses ‘completed some primary school’ and ‘completed primary school’ so these are not reported in table.

^b^Gross household income reported in Australian Dollars per annum, for all people in the household before tax, including all wages, salary, pensions and allowances. Low income < $60,000 AUD per annum, mid income = $60,001-$100,000 AUD per annum, high income > $100,000 AUD per annum.

^c^n = 36 missing responses for income; participants responses ‘refused to answer’ or ‘unsure’.

^d^SEIFA = Socioeconomic Index for Areas, area-level measure of SES using determined for postal code.

^e^Mother’s occupation coded into eight categories according to Australian and New Zealand Standard Classification of Occupations (ANZSCO). Additional category created for not in the labour force = individuals engaged in full-time home duties, retired persons, unemployed and students.

^f^n = 12 missing responses for mother’s occupation; participants did not provide sufficient information to enable accurate coding of occupation.

Children’s reports of usual serves of fruit and vegetables consumed each day largely fell short of Australian dietary recommendations, particularly for older children. In this study, 86.9% of boys and 88.7% of girls aged 9–11 years consumed at least 1–2 serves of fruit daily as per recommendation, and 35.6% of boys and 33.3% of girls aged 12–13 years met the guideline of 3–4 serves of fruit daily [33]. The vegetable intake recommendation of 3 serves daily for children aged 9–11 years was met by 42.4% of boys and 40.2% of girls. The recommendation of at least 4 serves of vegetables daily was met by 39.6% of boys and 40.9% of girls aged 12–13 years.

### Socioeconomic differences in dietary intake scores

Differences in dietary intake by SEP were found for all outcomes except girls’ healthy behaviours which were predicted only by marital status, with girls from two-parent families likely to engage in more healthy behaviours (Table 3). In general, mother’s education appeared to be the most consistent and strongest SEP predictor, and SEIFA was least frequently predictive of children’s dietary intake. In most instances, lower SEP was associated with poorer dietary outcomes, except for girls’ fruit intake. Overall the variance explained by socioeconomic factors was low, ranging from 0.5%-2.2% for healthy dietary intake outcomes (Table 3), and 0.6% - 4.9% for unhealthy dietary intake (Table 4). In the strongest model explaining the most variance, all five SEP factors, mother’s age and marital status explained 4.9% of variance in girls’ sweetened drink intake.

**Table 3:** Results of correlated component regression analyses for healthy dietary intake outcome measures

**BOYS (n = 275)**				**GIRLS (n = 353)**			
**Variable**	**CV predictor count**^**a**^	***β***	**Model goodness of fit indices**^**b**^	**Variable**	**CV predictor count**^**a**^	***β***	**Model goodness of fit indices**^**b**^
*Fruit intake* (*8 predictors*)				*Fruit intake* (*8 predictors*)			
SEIFA*	100	0.138	R^2^ = 0.052	Employment*	100	−0.122	R^2^ = 0.014
Mother’s education*	100	0.128	R^2^(CV) = 0.011	Child age*	89	−0.063	R^2^(CV) = 0.012
Employment*	96	−0.087	SD (CV) = 0.004	Mother’s occupation*	89	−0.063	SD (CV) = 0.009
Mother’s occupation*	92	0.067		Marital status*	82	0.033	
Marital status*	82	0.044		Mother’s education*	77	−0.010	
Mother’s age*	79	−0.040		Mother’s age*	71	−0.013	
Child age*	64	−0.035		SEIFA*	71	−0.002	
Income*	47	0.024		Income*	71	−0.006	
*Vegetable intake* (*1 predictor*)				*Vegetable intake* (*1 predictor*)			
Mother’s education*	90	0.120	R^2^ = 0.015	Employment*	83	−0.099	R^2^ = 0.010
SEIFA	67		R^2^(CV) = 0.007	Mother’s education	74		R^2^(CV) = 0.005
Employment	42		SD (CV) = 0.009	Mother’s occupation	10		SD (CV) = 0.003
Mother’s age	39			SEIFA	9		
Child age	22			Income	8		
Income	17			Mother’s age	7		
Mother’s occupation	2			Child age	5		
Marital status	1			Marital status	4		
*Healthy behaviours*^*C*^ (*4 predictors*)				*Healthy behaviours*^*C*^ (*1 predictors*)			
Employment*	100	−0.116	R^2^ = 0.036	Marital status*	54	0.077	R^2^ = 0.006
Marital status*	59	0.076	R^2^(CV) = 0.008	SEIFA	31		R^2^(CV) = 0.022
Mother’s occupation*	57	0.082	SD (CV) = 0.004	Child age	16		SD (CV) = 0.013
Mother’s education*	48	0.062		Mother’s age	12		
Income	12			Mother’s occupation	10		
SEIFA	3			Income	3		
Mother’s age	1			Employment	3		
Child age^^^	--			Mother’s education	1		

*Predictor retained in final model.

^^^Predictor not retained in any model.

*ß* = standardised regression coefficient.

^a^Cross-validation predictor count - Represents number of regressions in which predictor appeared. Predictor count of 100 indicates that predictor was present in all 100 regressions. Indicates importance of predictor together with standardised regression coefficient (*β*).

^b^Model goodness of fit indices: R^2^(CV) = cross-validated R^2^; SD (CV) = Standard deviation for cross-validated R^2^.

^c^Healthy behaviours: Breakfast intake, carrying water bottle, help parents with groceries, help to prepare dinner, eat dinner with the family.

**Table 4:** Results of correlated component regression analyses for unhealthy dietary intake outcome measures

**BOYS (n = 275)**				**GIRLS (n = 353)**			
**Variable**	**CV predictor count**^**a**^	***β***	**Model goodness of fit indices**^**b**^	**Variable**	**CV predictor count**^**a**^	***β***	**Model goodness of fit indices**^**b**^
*Non*-*core food intake* (*1 predictor*)				*Non*-*core food intake* (*1 predictor*)			
Mother’s education*	62	−0.111	R^2^ = 0.012	Mother’s education*	100	−0.119	R^2^ = 0.012
Employment	35		R^2^(CV) = 0.011	Marital status	22		R^2^(CV) = 0.006
Marital status	21		SD (CV) = 0.008	Employment	8		SD (CV) = 0.003
Mother’s age	20			Income	7		
Income	12			Mother’s occupation	6		
Child age	10			SEIFA	3		
SEIFA	10			Child age	2		
Mother’s occupation	10			Mother’s age	2		
*Sweetened drink intake* (*6 predictors*)				*Sweetened drink intake* (*7 predictors*)			
Child age*	90	0.087	R^2^ = 0.037	Income*	100	−0.085	R^2^ = 0.069
Mother’s occupation*	79	0.070	R^2^(CV) = 0.008	Mother’s education*	100	−0.063	R^2^(CV) = 0.049
SEIFA*	78	−0.070	SD (CV) = 0.005	Employment*	100	−0.067	SD (CV) = 0.005
Mother’s age*	71	−0.051		Mother’s age*	100	−0.066	
Income*	70	−0.057		SEIFA*	99	−0.064	
Employment*	66	−0.048		Mother’s occupation*	71	0.049	
Mother’s education	34			Marital status*	70	−0.045	
Marital status	32			Child age^	--		
*Unhealthy behaviours*^*C*^ (*4 predictors*)				*Unhealthy behaviours*^*C*^ (*3 predictors*)			
Child age*	100	0.128	R^2^ = 0.063	Mother’s education*	100	−0.184	R^2^ = 0.063
Mother’s education*	100	−0.121	R^2^(CV) = 0.037	Mother’s occupation*	100	−0.149	R^2^(CV) = 0.039
Mother’s occupation*	90	0.088	SD (CV) = 0.005	Employment*	100	−0.255	SD (CV) = 0.003
Income*	87	−0.084		Mother’s age	20		
Employment	3			Marital status	20		
SEIFA^^^	--			Child age	19		
Mother’s age^^^	--			Income	18		
Marital status^^^	--			SEIFA	3		

*Predictor retained in final model.

^^^Predictor not retained in any model.

*ß* = standardised regression coefficient.

^a^Cross-validation predictor count - Represents number of regressions in which predictor appeared. Predictor count of 100 indicates that predictor was present in all 100 regressions. Indicates importance of predictor together with standardised regression coefficient (*β*).

^b^Model goodness of fit indices: R^2^(CV) = cross-validated R^2^; SD (CV) = Standard deviation for cross-validated R^2^.

^c^Unhealthy behaviours: Eat dinner in front of TV, Eat snacks in front of TV, Eat fast food.

Fruit intake was predicted by all SEP variables for both genders. In boys, higher fruit intake was associated with living in a more advantaged area (higher SEIFA), higher maternal education, higher household income, employment in a lower status occupation and mothers not employed in the labour force. Girls had a higher fruit intake if mothers were employed in a higher status occupation or were not in the labour force, but also if their mothers had lower education attainment, they lived in a more disadvantaged area or had a lower household income. Higher vegetable intake was predicted by higher maternal education in boys, and mothers not employed in the labour force in girls. Engagement in more healthy eating behaviours in boys was predicted by mothers not employed in the labour force, employment in a lower status occupation and higher maternal education attainment.

Non-core food intake for boys and girls was predicted by mother’s education, with children of low educated mothers having higher non-core food intake. Boys consumed more sweetened drinks if mothers were employed in lower status occupations or not in the labour force, if they lived in more disadvantaged areas, and had a lower household income. Higher sweetened drink intake among girls was predicted by lower household income, lower education attainment, not being in the labour force, living in a more disadvantaged area and employment in a lower status occupation. Boys engaged in more unhealthy behaviours if their mothers had a lower education, were employed in a lower status occupation and had a lower income. For girls, more unhealthy behaviours were associated with lower maternal education, employment in a lower status occupation and not being in the labour force.

## Discussion

This study explored the role of five socioeconomic indicators in explaining socioeconomic differences in children’s diets, finding that each indicator was independently associated with at least one dietary outcome. In general, lower SEP was associated with poorer dietary behaviours, specifically higher intake of non-core foods, sweet drinks and more unhealthy behaviours; and lower intake of fruits, vegetables and less healthy behaviours. This is consistent with previous reports of socioeconomic gradients in children’s dietary intake [2-6,34,35]. Overall, the amount of variance explained by predictive CCR models was small, highlighting that a range of other factors are also important for children’s dietary intake.

However, this effect size is consistent with the magnitude of variance in children’s dietary intake explained by socio-demographic factors alone in other studies [36]. The dietary intake of children in this study did not meet suggested dietary targets, and the small differences in dietary intake between children of different socioeconomic groups suggest that all children, irrespective of SEP, would benefit from health promotion to improve dietary intake. However the necessity to target improvements in diets of low SEP children should not be dismissed. Socioeconomically-related differences in the diets of adults are generally larger than those identified for children [37,38], which may be attributed to declines to dietary quality during adolescence and therefore low SEP children are an important target group to improve eating habits before declines in dietary quality during adolescence and adulthood [39]. The effect of SEP appeared to be particularly important for girls’ sweetened drink intake, which was significantly and negatively associated with all measured SEP variables. This is in contrast to another study which found SEP predicted boys’, but not girls’ soft drink consumption [18]. A target area for future nutrition promotion may be encouraging the reduction of sweetened drink intake among low SEP children.

The effect of SEP differed between boys and girls, consistent with previous studies [4,18]. In particular, gender differences were identified in associations of fruit intake with SEP. In contrast to boys, fruit intake was higher among girls of low educated mothers, low income households and living in more disadvantaged neighbourhoods. This finding is difficult to explain and requires further investigation. Fruit, unlike vegetables, typically requires little preparation or cooking skills, and may be perceived as an easy to consume snack [40,41]. Low SEP women have reported lower levels of food preparation and cooking skills [42], and so might be more likely to provide fruit for a handy snack or part of a meal. If so, it is unclear why this was the case only for girls and not boys. Alternatively, these findings may be due to more general gender stereotypes around food, eating and health. From an early age, girls are socialised differently with regard to food and this may result in more health consciousness, more weight concerns, and different health beliefs and attitudes, which are translated into different dietary patterns [43-45]. Girls may perceive their food environments differently to boys, reporting more restrictions around eating than boys, despite parent reports indicating no differences in rule imposition between boys and girls [46]. However, it is unclear how SEP may impact on gender stereotypes around eating and this warrants future consideration.

Multiple socioeconomic factors independently predicted many of the dietary outcomes, suggesting the need to consider multiple socioeconomic indices when explaining gradients in children’s dietary intake. To provide further insight into potential covariance and conceptual overlap among SEP indicators, adjusted bivariate CCR regression models for each individual SEP indicator and dietary outcome were conducted (results available as Additional file 1: Table S1 online). The patterning of results between adjusted bivariate associations and multivariate models was similar, however there was attenuation or amelioration of associations between SEP and diet in some multivariate models compared with bivariate associations, suggesting some overlap between SEP variables. Turrell et al. [25] similarly found that in multivariate models, independent associations of SEP indicators with food purchasing changed compared with original bivariate associations, and suggested that this attenuation occurred as a result of unmeasured effects due to overlap between SEP indicators. However, correlations between SEP indicators are weak, and Turrell et al. [25] cautioned that researchers should not assume that each SEP indicator is tapping a similar underlying construct, and therefore indicators should not be used interchangeably. Correspondingly, the findings of this study highlight a risk of misrepresenting socioeconomic gradients if only a single measure of SEP is used. The differences between adjusted bivariate associations and multivariate models suggest that had results solely relied upon bivariate associations, the claims about SEP-diet indicators would have been misstated. Over- or underestimation of socioeconomic gradients in dietary intake may have implications for the development and implementation of dietary intervention strategies or health promotion campaigns. Distinct facets of SEP may influence children’s dietary intake by conceptually different pathways, and therefore inclusion of SEP variables in analyses should be driven by conceptual considerations. Further still, an individual’s SEP is defined by more than one SEP variable, and these variables may each influence dietary intake differently, but each may also mediate associations between other SEP indicators and diet. For example, education may influence occupation and income, which may then impact on diet; in this case occupation and income would be mediating variables. Formal tests of mediation were beyond the scope of this investigation, but future studies should consider the mediating role and interplay between different SEP variables in associations of SEP and children’s diet. We refer readers to Turrell et al. [25] which provides a detailed discussion of the stability and robustness of different SEP indicators in relation to adult diet. Given the evidence for the importance of each SEP indicator independently in relation to eating, in the following section we consider theoretical conceptual pathways of influence for SEP on children’s diet.

Mother’s education level was the most consistent predictor across all dietary outcomes. Low education was associated with higher intake of non-core foods and sweetened drinks, and more unhealthy behaviours, as well as lower vegetable intake and less healthy behaviours. Maternal education has been identified as the most consistent predictor of children’s diet, and low maternal education has been associated with poorer dietary intake across a range of dietary outcomes [1,3,5,23,34,47]. Higher education may enable individuals to better access, assimilate and put into practice health information [48,49], and may therefore better equip parents to understand and make use of health and nutrition information. Parents of lower education may have poorer nutrition knowledge [49,50], and consider health less often when making food choices for themselves and their children [48,51]. Conversely, parents of higher education may place more importance on healthy eating, which may be related with better nutrition knowledge [52]. Nutrition knowledge and health consideration may be related with healthier dietary intake among mothers and children, and may inform the types of foods parents provide for their family [49,51,53,54].

Findings for the effect of mother’s occupation and employment on children’s dietary intake were mixed. On the one hand, employment in higher status occupations was associated with more fruit intake and less sweetened drink intake, consistent with previous studies [18,19]. Unexpectedly, boys of mothers in higher status occupations reported less fruit intake and healthy behaviours and more unhealthy behaviours. This was explored further by considering the effect of maternal employment on children’s dietary intake, and we found that employment was associated with dietary intake independently of mother’s occupation. Children of employed mothers were likely to consume more sweetened drinks, fewer fruits and vegetables and engage in less healthy behaviours. Time spent in employment may impact on the time mothers have to engage in activities that can positively influence children’s food intake, such as supervising breakfast consumption, eating family meals and engaging children in food shopping. Time-poor mothers may be more reliant on takeaway meals, contributing to lower consumption of fruit and vegetables [1]. Neumark-Sztainer [55] reported that adolescents of unemployed mothers consumed family meals more frequently, and this was related to more positive dietary behaviours. Younger children of employed mothers had a higher sweetened drink intake and a lower likelihood of consuming fruits and vegetables as a snack [56]. Sweeting and West [23] found children of mothers in part-time employment to have a significantly lower risk for unhealthy eating, suggesting these mothers may have an economic advantage over homemakers and a time advantage over mothers who are employed full-time [23]. Occupation determines working conditions such as time spent in employment, flexibility of working arrangements and differential exposure to work place stressors, and these factors may affect children’s dietary intake [24,25]. Given this, it is likely that a combination of factors related to both occupation and employment influence children’s food intake.

This study found that higher income independently predicted more fruit intake and less sweetened drink intake and unhealthy behaviours. Income appeared less frequently as a predictor of children’s dietary intake than education, occupation and employment. This is consistent with previous literature, except that previous authors have also found income to be positively associated with vegetable consumption [1,28,35]. Income reflects the financial resources available for food purchasing, accessing resources and health professionals. Low income families may prioritise non-food expenses such as rent/mortgage, utility bills and school fees over food and health expenditure, as these expenses are less flexible [57], and must allocate a greater proportion of their overall income to purchasing food. Purchasing groceries according to dietary guidelines costs low income families 35-44% of their disposable income compared to approximately 20% for families of average income [57,58]. Purchasing patterns of low-income families indicate they may purchase fewer fruits and vegetables, foods high in fibre, low in fat, sugar and salt [59,60]. Healthy foods, particularly fruit and vegetables, may be perceived to be more expensive [61] and low-income adults are more likely to report price as a barrier to fruit and vegetable consumption [62,63].

SEIFA appeared least frequently as a predictor of dietary intake, with an effect of smaller magnitude compared with the other socioeconomic indicators. Living in a more disadvantaged area predicted higher sweetened drink intake and more unhealthy behaviours, and lower fruit intake for boys. The types of foods readily available for purchase in close proximity to the family home may influence children’s food consumption [64]. More access to convenience stores and fast food restaurants may contribute to higher intake of processed snack foods, displacing intake of fruits and vegetables [65,66]. However the evidence for disproportionate access to food stores by neighbourhood disadvantage is mixed. Studies conducted in the USA suggest that disadvantaged neighbourhoods may have fewer supermarkets and more convenience stores, resulting in higher prices and lower availability of healthy foods [67-71]. Evidence from Australian studies is less consistent, with some studies suggesting better access to supermarkets and greengrocers in more advantaged neighbourhoods [72,73], but other authors finding no difference in store availability between low and high SEP neighbourhoods [74]. Evidence of neighbourhood variation in takeaway and fast-food outlets is mixed, with studies showing no socioeconomic differences [75]; a higher density of fast-food outlets in low socioeconomic areas [76]; and conversely closer proximity to fast-food restaurants in more advantaged areas [73]. It is likely that the relationships between diet and neighbourhood are culturally and contextually specific, and may differ by region, state and country.

### Strengths and limitations of study

As this study was cross-sectional causality cannot be inferred, and although cross-sectional studies offer a snapshot of current associations, factors resulting in behaviour change cannot be identified. Individual SEP may predict participation in research, with lower participation rates among individuals of low SEP, and non-responders to dietary surveys may also differ from responders on dietary intake and attitudes [77,78]. This indicates a risk of a recruitment bias, whereby parents more ‘concerned’ about health and nutrition may have opted to participate which may have influenced the types of responses in questionnaires. However, parents were offered a $30 voucher as compensation for time taken to participate in the study, to encourage some parents to participate who may not have otherwise done so. There were no differences in scores reported for dietary intake between children who did and did not participate in phase two; however families who participated in phase two resided in neighbourhoods of higher SEP than those who did not. This potential respondent bias must be recognised as a limitation of this study, despite achieving a socioeconomically stratified participant sample and providing incentives for participation. Finally, the limitations of children reporting their own dietary intake need to be recognised, in terms of reporting errors and poorer recall of intake. However, the CNQ has been shown to have acceptable validity and reliability in children of this age group [27] and children of this age are capable of self-reporting dietary intake [79]. The strengths of this study include the relatively even distribution of the sample across socioeconomic strata. Multiple dimensions of SEP were considered simultaneously, allowing for independent and shared effects of demographic variables to be determined. Analyses were conducted separately for boys and girls, enabling the identification of sex-specific socioeconomic predictors of dietary intake. SEP data were not reduced into broad categories for analysis (i.e. low versus high), therefore increasing the sensitivity to detecting gradients across SEP strata. Online administration of the CNQ allowed for in-built measures, such as forced question response, to minimise missing data and reduce errors associated with data entry.

## Conclusions

Education, occupation/employment, income and to a lesser extent SEIFA were all associated with children’s dietary intake, in most situations (except girls’ fruit intake) with low SEP being associated with poorer dietary outcomes. Varying associations of SEP with diet were observed across different indicators of both SEP and diet, suggesting that different components of SEP may affect children’s eating differently, and therefore researchers should consider multiple SEP indicators when defining SEP in relation to children’s eating.

By conducting multivariate models, we were able to identify that education, occupation and income are all independently associated with various indices of children’s dietary intake, and that the nature of the association of SEP with diet differs according to children’s gender and the dietary outcome in question.

## Competing interests

The authors’ declare that they have no competing interests.

## Authors’ contributions

In addition to specific contributions listed below, all authors contributed to the study design, statistical analysis and interpretation of the data. DZ carried out data collection and drafted the manuscript. KB, NP and JD revised the manuscript. With the assistance of a statistician, DZ conceptualised and conducted the statistical analysis. All authors read and approved the final manuscript.

## Supplementary Material

Additional file 1: Table S1 Bivariate models of associations of socioeconomic indicators with children’s dietary intake. Click here for file
