# Supplementary material for: Describing socioeconomic gradients in children’s diets – does the socioeconomic indicator used matter?
Source: Int J Behav Nutr Phys Act. 2014 Mar 28;11:44. doi: 10.1186/1479-5868-11-44 (PMC3986827; doi:10.1186/1479-5868-11-44)
Supplement: Additional file 1: Table S1 — Bivariate models of associations of socioeconomic indicators with children’s dietary intake. [file 1479-5868-11-44-S1.docx]

**Additional file 1: Table S1**: Bivariate models of associations of socioeconomic indicators with children’s dietary intake

| **BOYS (n=275)** | | | | | | | **GIRLS (n=353)** | | | | | | |
| --- | --- | --- | --- | --- | --- | --- | --- | --- | --- | --- | --- | --- | --- |
| **Variable** | **CV predictor count^a^** | ***β*** | **Model goodness of fit indices^b^** | | | | | **Variable** | **CV predictor count^a^** | ***β*** | **Model goodness of fit indices^b^** | | |
|  |  |  | **R^2^** | **R^2^(CV)** | | **SD (CV)** | |  |  |  | **R^2^** | **R^2^(CV)** | **SD (CV)** |
| *Fruit intake* | | |  |  |  | | | *Fruit intake* | |  |  |  |  |
| Mother’s education* | 99 | 0.126 | 0.016 | 0.002 | 0.002 | | | Mother’s education | 28 | - | 0.004 | 0.004 | 0.004 |
| Mother’s occupation* | 74 | 0.043 | 0.007 | 0.019 | 0.009 | | | Mother’s occupation* | 93 | 0.044 | 0.009 | 0.007 | 0.012 |
| Mother’s employment* | 92 | -0.079 | 0.006 | 0.070 | 0.005 | | | Mother’s employment* | 97 | -0.099 | 0.010 | 0.002 | 0.002 |
| Income* | 98 | 0.048 | 0.010 | 0.001 | 0.001 | | | Income | 45 | - | 0.003 | 0.009 | 0.007 |
| SEIFA* | 99 | 0.052 | 0.020 | 0.009 | 0.004 | | | SEIFA | 5 | - | 0.004 | 0.008 | 0.007 |
| *Vegetable intake* | | |  |  |  | | | *Vegetable intake* | | |  |  |  |
| Mother’s education* | 98 | 0.120 | 0.014 | 0.003 | 0.002 | | | Mother’s education* | 100 | 0.087 | 0.010 | 0.001 | 0.001 |
| Mother’s occupation | 45 | - | 0.001 | 0.059 | 0.023 | | | Mother’s occupation | 3 | - | 0.004 | 0.012 | 0.008 |
| Mother’s employment* | 33 | -0.042 | 0.002 | 0.053 | 0.016 | | | Mother’s employment* | 100 | -0.101 | 0.010 | 0.003 | 0.002 |
| Income* | 92 | 0.078 | 0.006 | 0.006 | 0.005 | | | Income* | 45 | 0.025 | 0.001 | 0.053 | 0.028 |
| SEIFA* | 97 | 0.099 | 0.010 | 0.001 | 0.001 | | | SEIFA | 49 | - | 0.002 | 0.020 | 0.013 |
| *Healthy Behaviours ^C^* | | |  |  |  | | | *Healthy Behaviours ^C^* | | |  |  |  |
| Mother’s education* | 100 | 0.077 | 0.022 | 0.003 | 0.002 | | | Mother’s education | 9 | - | 0.012 | 0.001 | 0.002 |
| Mother’s occupation* | 100 | 0.123 | 0.036 | 0.011 | 0.004 | | | Mother’s occupation | 41 | - | 0.009 | 0.002 | 0.002 |
| Mother’s employment* | 100 | -0.143 | 0.044 | 0.016 | 0.006 | | | Mother’s employment | 3 | - | 0.016 | 0.002 | 0.002 |
| Income* | 55 | 0.040 | 0.015 | 0.001 | 0.002 | | | Income* | 96 | 0.043 | 0.010 | 0.001 | 0.002 |
| SEIFA | 14 | - | 0.018 | 0.002 | 0.002 | | | SEIFA* | 100 | 0.060 | 0.014 | 0.001 | 0.001 |
| *Non-core food intake* | | |  |  |  | | | *Non-core food intake* | | |  |  |  |
| Mother’s education* | 100 | -0.104 | 0.027 | 0.003 | 0.002 | | | Mother’s education* | 100 | -0.115 | 0.018 | 0.006 | 0.005 |
| Mother’s occupation* | 97 | 0.067 | 0.022 | 0.000 | 0.001 | | | Mother’s occupation* | 83 | 0.064 | 0.009 | 0.003 | 0.005 |
| Mother’s employment* | 100 | -0.099 | 0.027 | 0.002 | 0.002 | | | Mother’s employment | 13 | - | 0.005 | 0.006 | 0.006 |
| Income* | 100 | 0.053 | 0.018 | 0.001 | 0.001 | | | Income* | 90 | -0.115 | 0.016 | 0.004 | 0.002 |
| SEIFA* | 60 | 0.045 | 0.018 | 0.004 | 0.004 | | | SEIFA | 4 | - | 0.004 | 0.002 | 0.003 |
| *Sweetened drink intake* | | |  |  |  | | | *Sweetened drink intake* | | |  |  |  |
| Mother’s education | 0 | - | 0.026 | 0.010 | 0.003 | | | Mother’s education* | 100 | -0.134 | 0.051 | 0.032 | 0.004 |
| Mother’s occupation* | 100 | 0.098 | 0.034 | 0.009 | 0.003 | | | Mother’s occupation* | 100 | 0.120 | 0.047 | 0.026 | 0.003 |
| Mother’s employment* | 68 | -0.077 | 0.031 | 0.006 | 0.004 | | | Mother’s employment* | 100 | -0.130 | 0.049 | 0.030 | 0.004 |
| Income* | 92 | -0.081 | 0.033 | 0.009 | 0.006 | | | Income* | 100 | -0.256 | 0.063 | 0.042 | 0.004 |
| SEIFA* | 100 | -0.093 | 0.031 | 0.009 | 0.003 | | | SEIFA* | 100 | -0.116 | 0.042 | 0.024 | 0.002 |
| *Unhealthy behaviours ^d^* | | |  |  |  | | | *Unhealthy behaviours ^d^* | | |  |  |  |
| Mother’s education* | 100 | -0.153 | 0.054 | 0.034 | 0.005 | | | Mother’s education* | 100 | -0.191 | 0.037 | 0.028 | 0.002 |
| Mother’s occupation* | 100 | 0.151 | 0.049 | 0.029 | 0.003 | | | Mother’s occupation* | 100 | 0.100 | 0.022 | 0.006 | 0.003 |
| Mother’s employment | 10 | - | 0.027 | 0.016 | 0.002 | | | Mother’s employment* | 100 | -0.174 | 0.030 | 0.022 | 0.004 |
| Income* | 100 | -0.130 | 0.045 | 0.022 | 0.006 | | | Income* | 100 | -0.073 | 0.017 | 0.001 | 0.001 |
| SEIFA | 0 | - | 0.029 | 0.014 | 0.003 | | | SEIFA* | 73 | -0.039 | 0.016 | 0.001 | 0.001 |
|  |  |  |  |  |  | | |  |  |  |  |  |  |

All models control for child age, mother’s age and marital status.

* Predictor retained in final model (CCR employs cross-validation to produce a linear combination of explanatory variables used to predict the dependent variable, and therefore not all variables entered into CCR analysis are retained in the final predictive models).

*ß* = standardised regression coefficient.

^a^ Cross-validation predictor count - Represents number of regressions in which predictor appeared. Predictor count of 100 indicates that predictor was present in all 100 regressions. Indicates importance of predictor together with standardised regression coefficient (*β*).

^b^ Model goodness of fit indices: R^2^(CV) = cross-validated R^2^; SD (CV) = Standard deviation for cross-validated R^2.^

^c^ Healthy behaviours: Breakfast intake, carrying water bottle, help parents with groceries, help to prepare dinner, eat dinner with the family.

^d^ Unhealthy behaviours: Eat dinner in front of TV, Eat snacks in front of TV, Eat fast food.
